# Case Report: Intracranial growing teratoma syndrome in two children with suprasellar mixed germ cell tumors: implications for the timing of surgery

**DOI:** 10.3389/fonc.2026.1871160

**Published:** 2026-07-31

**Authors:** Qizi Wu, Jian Li, Jing Ge, Lei Wu, Tao Li, Li Zhou

**Affiliations:** 1 Department of Hematology, Nanjing Drum Tower Hospital, Affiliated Hospital of Medical School, Nanjing University, Nanjing, China; 2 Department of Oncology, Children’s Hospital of Nanjing Medical University, Nanjing, China; 3 Department of Hematology, Children’s Hospital of Nanjing Medical University, Nanjing, China

**Keywords:** chemotherapy, intracranial growing teratoma syndrome, mixed germ cell tumor, panhypopituitarism, suprasellar tumor, surgery

## Abstract

**Background:**

Intracranial growing teratoma syndrome (iGTS) is characterized by paradoxical tumor enlargement despite normalization of tumor markers during treatment of germ cell tumors. Its recognition is critical, particularly in the suprasellar region where mass effect can lead to significant morbidity.

**Case presentation:**

We report two pediatric patients with suprasellar mixed germ cell tumors treated with alternating etoposide–cisplatin and ifosfamide–etoposide chemotherapy. In both cases, serum alpha-fetoprotein (AFP) and beta-human chorionic gonadotropin (β-HCG) levels declined markedly during treatment; however, clinical deterioration and radiologic tumor enlargement occurred. Surgical resection revealed mature teratoma in both cases, confirming classic iGTS after histopathologic review. Gross total resection was achieved in both cases, followed by completion of chemotherapy and craniospinal irradiation. At follow-up, both patients remained in remission but developed permanent hypothalamic–pituitary dysfunction.

**Results:**

These cases illustrate a clear dissociation between biochemical response and local mass enlargement, consistent with classic iGTS in both patients.

**Conclusion:**

Declining tumor markers should not be interpreted as reassurance in the presence of worsening symptoms or tumor enlargement. Early surgical intervention should be considered in selected patients to relieve mass effect and potentially reduce long-term morbidity.

## Introduction

Intracranial germ cell tumors are uncommon neoplasms primarily affecting children and teenagers aged 10 to 20 years, with a yearly incidence rate of 0.17 per 100,000 individuals (1). These tumors are more prevalent in Asians, comprising 8% to 14% of all pediatric central nervous system tumors, which is significantly higher than the 0.5% to 3% incidence in Europe and the United States (2, 3). The tumors typically arise in the midline area, with approximately 75% located in the pineal region and 10% to 20% in the suprasellar region.

Intracranial growing teratoma syndrome (iGTS) is a relatively rare complication that occurs during the treatment of intracranial germ cell tumors. Growing teratoma syndrome was first described by Logothetis et al. in extracranial germ cell tumors (4). In intracranial germ cell tumors, the reported frequency of iGTS is approximately 5%–6.5% (5, 6). The strict diagnosis of classic iGTS requires meeting three conditions: normalization or marked decline of alpha-fetoprotein (AFP) and/or beta-human chorionic gonadotropin (β-HCG), paradoxical enlargement of the lesion during or after therapy, and histologic confirmation of mature teratoma without viable malignant germ cell tumor components.

Here, we describe two children with suprasellar mixed germ cell tumors who developed iGTS during treatment and discuss the implications for the timing of surgery.

## Case presentation

A summary of the treatment timeline, serum tumor marker trends, imaging findings, surgical management, and outcomes for both patients is provided in **Table 1**.

**Table 1:** Clinical timeline of two patients.

Case	Time point	Clinical status	Serum AFP (ng/mL)	Serum β-HCG (mIU/mL)	CSF AFP (ng/mL)	CSF β-HCG (mIU/mL)	Endocrine status	MRI/Key findings	Intervention
Case 1	Diagnosis	Headache, blurred vision, weakness, hydrocephalus	187.3	466.3	<0.605	2.24	Central diabetes insipidus only	33×31×30 mm suprasellar cystic-solid mass	VP shunt; start EP/IE
	Before cycle 2	Clinical improvement	125	6.69	–	–	–	No major interval concern reported	Continue chemotherapy
	Before cycle 3	Biochemical response	75	0.281	–	–	–	—	Continue chemotherapy
	After 2 cycles	Recurrent headache/vomiting	5.8	<0.1	–	–	–	Tumor enlarged to 40×35×34 mm	Tumor resection (classic iGTS)
	Postoperative	Stable	1.44	<0.1	–	–	Panhypopituitarism developed/no pituitary stalk transection documented	No residual tumor	Complete chemotherapy + CSI
	~28-month follow-up	Remission	Normal	Normal	–	–	Panhypopituitarism, mild hypernatremia, temperature dysregulation	No recurrence	Hormone replacement
Case 2	Diagnosis	Somnolence, vomiting	379.8	2.19	41.72	2.67	Hypothyroidism only	36×35×45 mm suprasellar mass	Start EP/IE
	First chemotherapy cycle	Neurologic deterioration	41.72	—	–	–	–	Hydrocephalus progression	VP shunt
	During first cycle	Persistent symptoms	Declining	2.67	–	–	–	Slight enlargement despite marker decline	Tumor resection
	Postoperative/ongoing therapy	Recovery	3.97 then <0.60	<0.1	–	–	Panhypopituitarism developed/no pituitary stalk transection documented	No residual tumor	Complete chemotherapy + CSI
	~27-month follow-up	Remission	Normal	Normal	–	–	Panhypopituitarism, mild hypernatremia, temperature dysregulation	No recurrence	Hormone replacement

### Case 1

**Figure 1:**
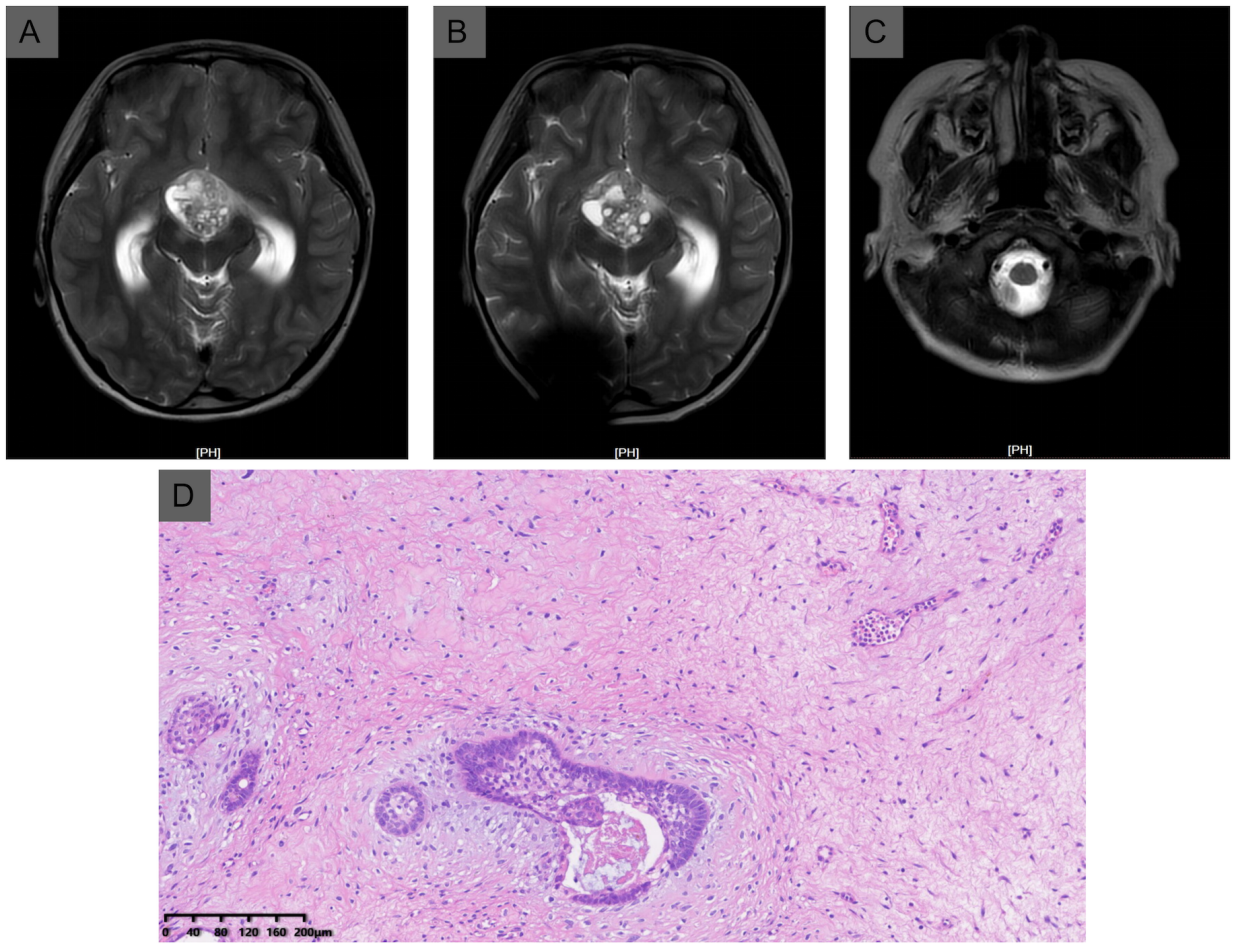
MRI findings and pathology in Case 1. **(A)** Initial MRI showing a heterogeneous suprasellar mass with cystic components, pituitary involvement, and compression of the optic pathway. **(B)** Post-treatment MRI revealing interval enlargement of the mass despite a biochemical response. **(C)** Postoperative MRI after gross total resection showing no residual tumor. **(D)** Pathological examination after surgical resection confirming mature teratoma without malignant elements.

A 10-year-3-month-old girl presented with persistent low-grade fever, intermittent headache, blurred vision, and limb weakness. Baseline visual assessment showed subjective visual blurring; formal visual field testing was not available. At diagnosis, serum AFP was 187.3 ng/mL (reference range, 0.6–7 ng/mL) and β-HCG was 466.3 mIU/mL (reference range, 0–5.3 mIU/mL). CSF AFP and CSF β-HCG were <0.605 ng/mL and 2.24 mIU/mL, respectively. Brain magnetic resonance imaging (MRI) demonstrated a heterogeneous suprasellar mass measuring 33 × 31 × 30 mm with cystic components, heterogeneous enhancement, pituitary involvement, obscuration of the posterior pituitary bright spot and stalk, and compression of the optic pathway (**Figure 1A**). Baseline endocrine evaluation showed central diabetes insipidus, whereas no other definite anterior pituitary hormone deficiency was documented at diagnosis. Obstructive hydrocephalus was present. Initial staging evaluation showed no evidence of cerebrospinal fluid (CSF) dissemination. Given the markedly elevated serum AFP and β-HCG levels together with the characteristic midline suprasellar location, a marker-based diagnosis of nongerminomatous germ cell tumor (NGGCT) was considered sufficient for treatment initiation. Upfront biopsy was deferred because the lesion involved the suprasellar/third ventricular region, where surgical intervention was considered to carry substantial risks of hypothalamic, pituitary, and visual morbidity.

Following initial evaluation, a ventriculoperitoneal shunt was placed, with improvement in symptoms of intracranial hypertension. Systemic chemotherapy consisting of alternating etoposide–cisplatin (EP) and ifosfamide–etoposide (IE) cycles was initiated according to our institutional pediatric CNS NGGCT protocol, which is based on platinum–etoposide–ifosfamide regimens used in international cooperative group studies and contemporary Chinese pediatric CNS GCT treatment experience (7). Before cycle 2, AFP decreased to 125 ng/mL and β-HCG decreased to 6.69 mIU/mL. Before cycle 3, AFP further declined to 75 ng/mL and β-HCG declined to 0.281 mIU/mL.

After two cycles of chemotherapy, she was readmitted with recurrent headache and vomiting. Visual symptoms persisted during this episode. At that time, AFP had decreased to 5.8 ng/mL and β-HCG had decreased to <0.1 mIU/mL, indicating near-complete biochemical response. However, repeat MRI demonstrated interval enlargement of the lesion to 40 × 35 × 34 mm, with persistent heterogeneous signal and multiple cystic components causing ongoing mass effect (**Figure 1B**). Given the discordance between biochemical response and tumor enlargement, tumor resection was performed shortly thereafter.

Intraoperatively, the third ventricle was filled with tumor. The lesion was gray-white, firm, moderately vascular, and well circumscribed, with multiple cystic areas containing clear fluid. The inferior surface of the tumor was closely adherent to the pituitary stalk and hypothalamic region; careful dissection was performed without intentional transection of the pituitary stalk. Gross total resection was achieved. Early postoperative MRI (**Figure 1C**) showed no residual tumor. After resection, her visual function gradually improved. Postoperatively, AFP was 1.44 ng/mL and β-HCG was <0.1 mIU/mL. Histopathologic examination demonstrated mature teratoma composed of well-differentiated teratomatous elements, without primitive neuroepithelial tissue or viable malignant non-teratomatous germ cell tumor components, confirming classic iGTS (**Figure 1D**).

The patient subsequently completed six cycles of alternating EP/IE chemotherapy, followed by craniospinal irradiation (30 Gy) with a tumor-bed boost to 54 Gy. At the last follow-up, approximately 28 months after diagnosis, she remained in remission with no radiologic evidence of recurrence, and ophthalmologic assessment showed normalization of visual function. However, compared with baseline endocrine status, which was limited to central diabetes insipidus, she had developed panhypopituitarism accompanied by mild hypernatremia and persistent temperature dysregulation, requiring long-term hormone replacement including hydrocortisone, levothyroxine, and desmopressin. Because the pituitary stalk was not transected, endocrine deterioration was considered more likely related to hypothalamic–pituitary involvement by the tumor, surgical manipulation of the suprasellar/third ventricular region, postoperative local effects, and subsequent irradiation.

### Case 2

**Figure 2:**
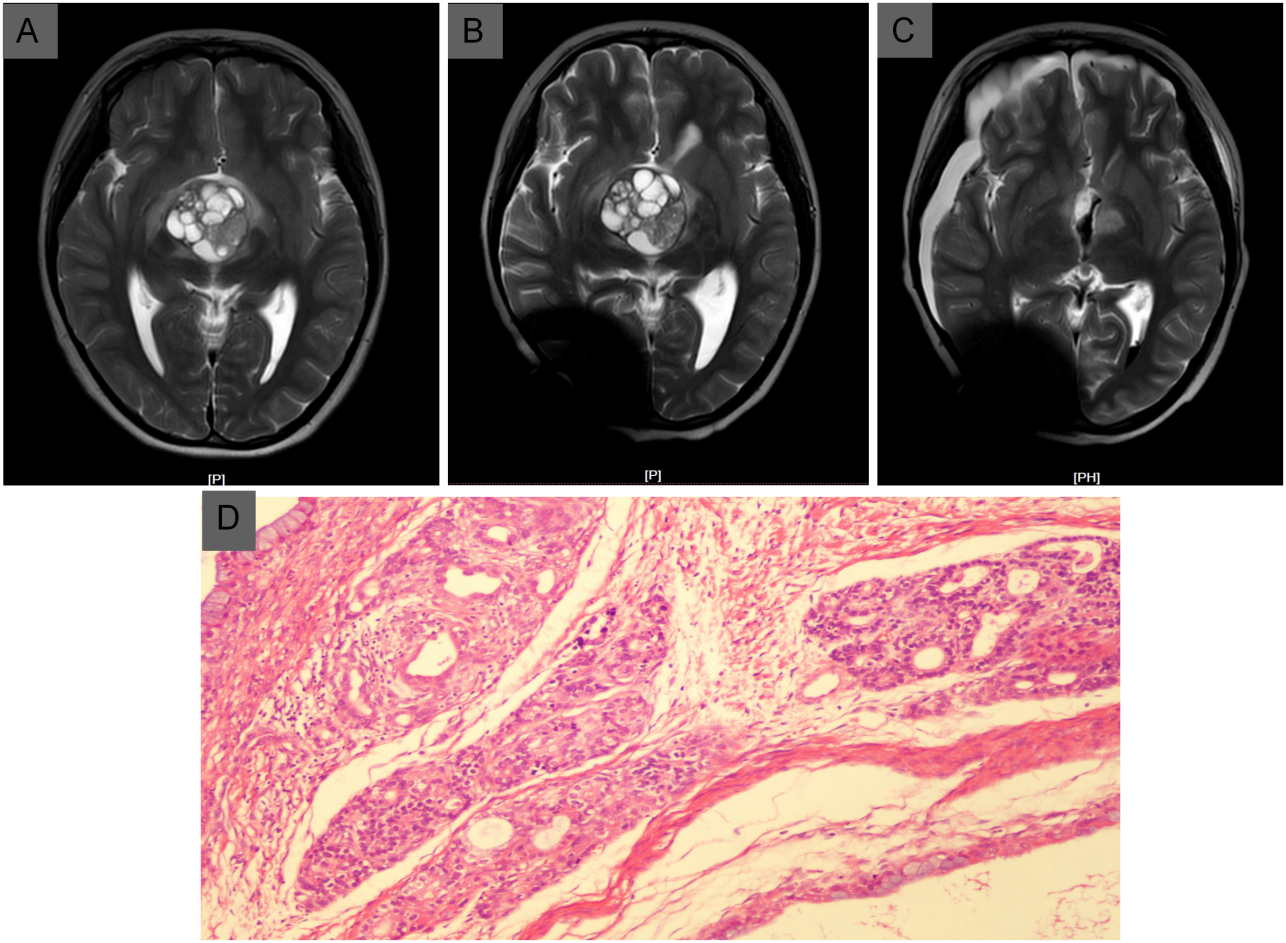
MRI findings and pathology in Case 2. **(A)** Initial MRI showing a heterogeneous suprasellar mass with cystic components, diffusion restriction, and marked enhancement of the solid component. **(B)** Follow-up MRI showing slight interval enlargement of the tumor and persistent ventricular dilatation. **(C)** Postoperative MRI after gross total resection showing no residual tumor and improvement in ventricular dilatation. **(D)** Low-power hematoxylin and eosin staining showing predominantly mature teratomatous architecture with focal epithelial and mesenchymal elements displaying limited developmental immaturity. No primitive neuroepithelial or neural tube-like elements were identified on histopathologic re-review.

An 8-year-7-month-old girl presented with lethargy, somnolence, frequent vomiting, and decreased visual acuity in the left eye on initial examination. Brain MRI at diagnosis revealed a heterogeneous suprasellar cistern mass measuring 36 × 35 × 45 mm, with multiple cystic components, focal diffusion restriction, surrounding edema, and marked enhancement of the solid component (**Figure 2A**). Serum AFP was 379.8 ng/mL and β-HCG was 2.19 mIU/mL. CSF AFP and CSF β-HCG were 41.72 ng/mL and 2.67 mIU/mL, respectively. CSF cytology showed no evidence of malignant dissemination. Baseline endocrine evaluation showed central hypothyroidism without documented diabetes insipidus or other definite pituitary hormone deficiencies at diagnosis. Because of the markedly elevated serum AFP level and the typical radiologic appearance of a midline suprasellar germ cell tumor, treatment was initiated without prior biopsy. In addition, the large size of the lesion and its close relationship to critical hypothalamic–pituitary structures made upfront surgical intervention high risk.

Given the high surgical risk at presentation, the patient received the same chemotherapy regimen as Case 1 shortly after diagnosis. AFP decreased rapidly during the first week of chemotherapy, reaching 41.72 ng/mL. Despite early biochemical response, the patient developed progressive neurologic deterioration and became severely somnolent. Because of impaired consciousness and worsening hydrocephalus, formal visual acuity and visual field assessment could not be reliably repeated at the time of clinical deterioration. Emergency computed tomography demonstrated marked ventricular enlargement consistent with obstructive hydrocephalus, and urgent ventriculoperitoneal shunting was performed. However, her level of consciousness did not improve. Repeat MRI during the first chemotherapy cycle demonstrated slight interval enlargement of the tumor with persistent ventricular dilatation (**Figure 2B**). Preoperative β-HCG was 2.67 mIU/mL.

Given the dissociation between declining tumor markers and radiologic progression with clinical worsening, iGTS was suspected clinically. Tumor resection was performed shortly thereafter.

Intraoperatively, the third ventricle was occupied by tumor. The lesion had a smooth surface, firm consistency, moderate vascularity, and cystic degeneration with clear fluid. It was well circumscribed without obvious adhesion to adjacent thalamic structures. Gross total resection was achieved. Postoperative MRI showed no residual tumor and improvement in ventricular dilatation (**Figure 2C**). Histopathologic examination demonstrated a predominantly mature cystic–solid teratoma composed of well-differentiated teratomatous elements (**Figure 2D**). Focal epithelial and mesenchymal areas showed limited developmental immaturity; however, histopathologic re-review by senior pathologists did not identify primitive neuroepithelial or neural tube-like elements, or any viable malignant germ cell tumor components. Therefore, the lesion was interpreted as mature teratoma rather than immature teratoma, fulfilling the histopathologic criteria for classic iGTS.

Following surgery and continued chemotherapy, serum AFP decreased to 3.97 ng/mL and subsequently normalized (<0.60 ng/mL); β-HCG remained <0.1 mIU/mL thereafter. After resection and improvement of hydrocephalus, the decreased visual acuity in the left eye gradually recovered.

She subsequently completed six cycles of alternating EP/IE chemotherapy, followed by craniospinal irradiation (30 Gy) with a tumor-bed boost to 54 Gy. At the last follow-up, approximately 27 months after diagnosis, she remained in remission with no radiologic evidence of recurrence, and the decreased visual acuity in the left eye gradually recovered, and visual function returned to normal at follow-up. Compared with baseline endocrine status, which showed hypothyroidism only, she had developed panhypopituitarism accompanied by mild hypernatremia and persistent temperature dysregulation, requiring long-term hormone replacement therapy. No definite pituitary stalk transection was documented; therefore, endocrine deterioration was considered multifactorial, related to pre-existing suprasellar involvement, surgical manipulation, postoperative local effects, and subsequent irradiation.

Molecular profiling was not performed in either case because it was not routinely available in our clinical workflow at the time of treatment. This limitation was considered relevant because histopathologic re-review was required to clarify the distinction between mature teratoma with limited developmental immaturity and definitive immature teratoma in Case 2.

## Discussion

iGTS is a rare but clinically important phenomenon observed in children and adolescents treated for NGGCTs, especially mixed germ cell tumors with teratomatous components. Originally described by Logothetis et al. in extracranial germ cell tumors, classic iGTS is characterized by paradoxical enlargement of the tumor mass despite normalization or marked decline of tumor markers, with mature teratoma identified at resection (4, 5). In our two suprasellar cases, AFP and β-HCG declined markedly during chemotherapy, whereas the tumors enlarged and caused progressive mass effect. Surgical resection confirmed mature teratoma in both cases after histopathologic review. These findings highlight that radiologic and clinical progression should not be dismissed when tumor markers are improving.

The pathogenesis of iGTS remains incompletely understood. The most widely accepted hypothesis is that chemotherapy eradicates chemosensitive malignant germ cell tumor components, whereas mature teratomatous elements persist and enlarge. Chemotherapy-induced maturation and expansion of cystic components within mature teratoma have also been proposed (8, 9). Radiologically, iGTS is typically suggested by enlargement of a cystic–solid or multicystic mass despite declining or normalized tumor markers (6, 8, 10). Our cases followed this pattern: Case 1 enlarged from 33 × 31 × 30 mm to 40 × 35 × 34 mm after two cycles of chemotherapy, and Case 2 showed persistent cystic–solid enlargement with hydrocephalus and neurologic deterioration despite rapid AFP decline. Although CNS germ cell tumors are more common in East Asian populations, current evidence does not demonstrate that geography independently increases the risk of iGTS.

The main clinical implication of these cases concerns surgical timing. In marker-positive suprasellar NGGCTs, treatment may reasonably begin without upfront biopsy when serum and CSF markers and imaging strongly support the diagnosis. This approach was used in both patients because of the risks of operating near the hypothalamic–pituitary axis and optic pathway. However, our cases show that early clinical deterioration or cystic–solid enlargement during biochemical response should prompt surgical reassessment. Surgery is both diagnostic and therapeutic: it confirms histopathology, relieves mass effect, and permits appropriate continuation of germ cell tumor-directed therapy. In the North American and Australian series, all patients underwent resection, gross total resection was achieved in 79%, and 95% were alive at a median follow-up of 5.3 years (5). Representative reports and series relevant to the diagnosis, imaging features, surgical management, recurrence, and follow-up of iGTS are summarized in **Table 2**. Therefore, surgical decision-making should integrate symptoms and imaging findings, rather than rely solely on serum or CSF tumor marker trends.

**Table 2:** Representative reports and series of intracranial growing teratoma syndrome.

Study	Patients/setting	Radiology/pathology at iGTS	Management and outcome	Relevance to current report
Kong et al., 2009 (8) J Neurosurg Pediatr	Institutional series focused on iGTS mimicking tumor relapse after treatment of intracranial GCT.	Enlarging residual mass can mimic relapse; pathology confirmed mature teratomatous tissue in iGTS lesions.	Second-look surgery was emphasized to distinguish iGTS from relapse and guide further treatment.	Supports the need to avoid interpreting radiologic growth with falling markers as malignant progression alone.
Kim et al., 2011 (6) J Neurooncol	Retrospective cohort of 170 intracranial GCTs; 11 iGTS cases (6.5% overall; 21% among NGGCTs).	All 11 showed honeycomb-shaped multicystic growth on MRI; resected lesions showed teratomatous components.	Surgery in all; complete excision in 9. Eight alive without recurrence; two with residual masses died from iGTS progression.	Provides classic radiologic signature and supports timely radical excision when feasible.
Oya et al., 2014 (9) Childs Nerv Syst	Two pineal mixed GCT cases plus literature review focused on pathogenesis.	Authors questioned whether rapid growth reflects cellular proliferation or expansion of multiple cysts within mature teratoma.	Surgical pathology and low proliferative activity supported the hypothesis that cyst expansion may contribute to rapid enlargement.	Useful for discussing pathogenesis and why multicystic enlargement may occur during biochemical response.
Glass et al., 2014 (13) Childs Nerv Syst	Two pediatric iNGGCT cases with GTS followed by secondary malignant transformation.	Growing lesions initially showed benign teratoma; later transformation included malignant rhabdomyosarcoma and malignant carcinoid tumor.	Partial resection and radiotherapy were part of management; authors emphasized risk of malignant transformation of residual teratoma.	Supports discussing malignant transformation as a rare but clinically important differential diagnosis.
Michaiel et al., 2020 (5) J Neurooncol	International series from 22 North American/Australian centers; 39 iGTS cases among 777 pediatric CNS GCTs (5%).	Paradoxical growth during treatment despite marker normalization or decline; mature teratoma was the dominant surgical pathology.	All underwent resection; gross total resection in 79%; 95% alive at median 5.3-year follow-up, including 5 with stable residual mass.	Largest Western series; supports frequency estimate, risk features, and surgery as the mainstay of treatment.
Tsuyuguchi et al., 2020 (10) World Neurosurg	Institutional CNS-NGGCT series and literature review; 16 CNS-NGGCT patients treated, 6 primary GTS and 1 recurrent GTS described.	Literature review found most primary GTS cases consisted of enlargement with multicystic components.	Salvage surgery used during chemo/chemoradiotherapy; previous primary GTS may be a risk factor for recurrent GTS.	Directly addresses recurrent GTS, multicystic imaging, and need for surveillance.
Tasaka et al., 2021 (16) J Pediatr Hematol Oncol	Single NGGCT case with very slow intracranial GTS growth and intraventricular lipid accumulation.	Intraventricular lipid accumulation and indolent lesion growth highlighted clinicopathologic heterogeneity.	Observation and long-term radiologic follow-up were emphasized because of asymptomatic slow growth.	Supports long-term surveillance even after apparent tumor control.
Satake et al., 2024 (12) Current Oncology	Single pediatric pineal mixed GCT case with suspected iGTS and malignant features.	Resection showed teratoma with malignant features; molecular analysis identified DMRT1 loss and 12p gain.	Radical resection followed by multimodal therapy; authors discussed possible molecular drivers of malignant phenotype.	Supports molecular discussion and highlights rare malignant features as an important differential consideration in teratomatous lesions.
Hsieh et al., 2023 (15) Cancer Medicine	Pediatric GTS series plus literature review: 5 institutional cases plus 93 literature cases (98 total); 52 intracranial MGCTs.	GTS defined by enlarging masses after chemotherapy with normal markers and mature teratoma on pathology.	Most pediatric patients survived; 95/98 were alive in the compiled cohort.	Provides pediatric context and supports need for long-term outcome and recurrence assessment.
Schultz et al., 2015 (18) Pediatr Blood Cancer	Single child with large, rapidly growing unresectable pineal region GTS.	Solid enhancing components were followed radiologically.	PD0332991/palbociclib was administered and stabilized the solid enhancing components of the mass.	Supports cautious mention that targeted therapy such as CDK4/6 inhibition remains investigational for unresectable iGTS.

AFP, alpha-fetoprotein; beta-HCG, beta-human chorionic gonadotropin; CNS, central nervous system; CSF, cerebrospinal fluid; GCT, germ cell tumor; GTS, growing teratoma syndrome; iGTS, intracranial growing teratoma syndrome; MGCT, malignant germ cell tumor; NGGCT, nongerminomatous germ cell tumor; NR, not reported in accessible abstract; RT, radiotherapy.

Classic iGTS should be distinguished from progressive or recurrent NGGCT, immature teratoma, and malignant transformation. The key diagnostic features of classic iGTS are declining or normalized tumor markers, paradoxical enlargement of the mass, and histologic confirmation of mature teratoma without viable malignant germ cell tumor components. According to the WHO classification framework, immature teratoma requires definite immature tissue elements, particularly primitive neuroepithelial or neural tube-like components (11). In Case 2, histopathologic re-review showed predominantly mature teratomatous tissue with focal developmentally less mature epithelial and mesenchymal areas, but no primitive neuroepithelial or neural tube-like elements and no viable malignant germ cell tumor components. Therefore, the lesion was interpreted as mature teratoma, fulfilling the histopathologic criteria for classic iGTS. Malignant transformation is rare but should be considered when tumor growth is unusually aggressive or when somatic-type malignant components are identified (12, 13). Molecular profiling was not performed in our patients; however, published genomic analyses of intracranial teratomas suggest that molecular studies may help clarify difficult cases in the future (14).

Functional morbidity is particularly important in suprasellar iGTS. Both patients had endocrine dysfunction at diagnosis, but each was initially limited to one manifestation: central diabetes insipidus in Case 1 and hypothyroidism in Case 2. Both later developed panhypopituitarism despite no documented pituitary stalk transection, suggesting multifactorial injury related to tumor involvement, mass effect, surgical manipulation, postoperative local effects, and irradiation. Visual symptoms improved after decompression in both cases. Long-term follow-up should therefore include MRI surveillance as well as endocrine and ophthalmologic assessment. Recurrence is generally managed surgically when feasible, whereas unresectable or subtotally resected disease requires individualized management; targeted approaches such as CDK4/6 inhibition remain investigational (10, 15–18).

This report has limitations, including its retrospective design, small sample size, and lack of molecular profiling. Nevertheless, the two cases illustrate a practical diagnostic and therapeutic dilemma in suprasellar NGGCTs: improving tumor markers do not exclude clinically significant teratomatous enlargement, particularly when progressive mass effect develops during therapy.

## Conclusion

In children with suprasellar mixed germ cell tumors, declining AFP and β-HCG levels should not be interpreted as reassurance when neurologic, visual, or hypothalamic–pituitary symptoms worsen or when interval tumor enlargement is observed on imaging. Such discordance should raise suspicion for iGTS. Timely surgical resection is critical for relieving mass effect, establishing definitive histopathologic diagnosis, and guiding subsequent therapy. Earlier surgical intervention should be considered in selected high-risk patients with cystic–solid suprasellar lesions and progressive mass-effect symptoms during biochemical response.

## Data Availability

The original contributions presented in the study are included in the article. Further inquiries can be directed to the corresponding authors.
